# Porous three-dimensional graphene foam/Prussian blue composite for efficient removal of radioactive ^137^Cs

**DOI:** 10.1038/srep17510

**Published:** 2015-12-16

**Authors:** Sung-Chan Jang, Yuvaraj Haldorai, Go-Woon Lee, Seung-Kyu Hwang, Young-Kyu Han, Changhyun Roh, Yun Suk Huh

**Affiliations:** 1 Department of Biological Engineering, Biohybrid Systems Research Center (BSRC), Inha University, 100, Inha-ro, Incheon, 402-751, Korea; 2 Biotechnology Research Division, Advanced Radiation Technology Institute (ARTI), Korea Atomic Energy Research Institute (KAERI), 29, Geumgu-gil, Jeongeup, Jeonbuk, 580-185, Korea; 3 Department of Energy and Materials Engineering, Dongguk University, 30, Pildong-ro 1-gil, Seoul, 100-715, Korea; 4 Quality Management Team, Korea Institute of Energy Research, 152, Gajeong-ro, Daejeon, 305-343, Korea; 5 Radiation Biotechnology and Applied Radioisotope Science, University of Science Technology (UST), 217 Sinsung-Dong, Yu-Seong Gu, Daejeon, Korea

## Abstract

In this study, a simple one-step hydrothermal reaction is developed to prepare composite based on Prussian blue (PB)/reduced graphene oxide foam (RGOF) for efficient removal of radioactive cesium (^137^Cs) from contaminated water. Scanning electron microscopy and transmission electron microscopy show that cubic PB nanoparticles are decorated on the RGO surface. Owing to the combined benefits of RGOF and PB, the composite shows excellent removal efficiency (99.5%) of ^137^Cs from the contaminated water. The maximum adsorption capacity is calculated to be 18.67 mg/g. An adsorption isotherm fit-well the Langmuir model with a linear regression correlation value of 0.97. This type of composite is believed to hold great promise for the clean-up of ^137^Cs from contaminated water around nuclear plants and/or after nuclear accidents.

Water pollution and associated environmental issues have become a hot topic in recent years. Rapid industrialization has led to massive increase in the amount of wastewater that industries discharge into the environment. Water pollution is caused by oil spill, heavy metals, dyes, and organic compounds released by industries, as well as *via* unpredictable accidents. As a result of the earthquake on March 11, 2011, and the subsequent tsunami, water levels as high as 15 m inundated nuclear power plants, causing a loss of power and subsequent disruption of control and cooling system failure^1^. Additionally, it was estimated that thousands of tons of radionuclide contaminated water leaked into the sea after the Fukushima Daiichi nuclear disaster, including the major radionuclide activity of 940 TBq of radioactive cesium (^137^Cs). The radionuclide of ^137^Cs, which has a half-life of 30 years, is hazardous as it exerts toxic effects *via* beta-particles and strong gamma rays. Contaminated water requires millions of years to recoup; therefore, a great deal of effort has been directed toward eliminating these radioactive elements^2^. The removal of ^137^Cs from contaminated water has become an emerging issue since the Fukushima Daiichi Nuclear Power Plant disaster^3^.

Graphene, a paper-like monolayer of sp^2^-bonded carbon atoms patterned in a hexagonal lattice has recently attracted a great deal of attention. One atom thick graphene has high thermal conductivity, high electrical conductivity, high room temperature carrier mobility, lateral quantum confinement, etc.^4^,^5^. The material has prompted enormous scientific interest owing to its excellent electronic capacity, mechanical properties, superior chemical stability, and high specific surface area. These peculiar properties have highlighted the potential of this material in a variety of applications, such as electronics^6^, sensors^7^, catalysis^8^, energy-storage devices^9^, and drug delivery^10^. Recently, graphene-inorganic composite materials, derived from the integration of graphene oxide (GO) sheets with functional inorganic nanomaterials, have been intensively developed^11^,^12^,^13^. The composite materials lead to excellent properties for various potential applications^14^,^15^, due to synergistic effects.

Many adsorbent materials including inorganic adsorbents^16^, biomass^17^, clay minerals^18^, and metal oxides^19^ have been previously investigated for the ability to remove ^137^Cs from contaminated water. However, these adsorbents are either difficult to synthesize or too expensive for large-scale application. Prussian blue (PB, ferric hexacyanoferrate) is a face-centered cubic lattice, zeolite-like inorganic material that exchanges its potassium ions for cesium ions because of its high affinity to Cs in solution^19^,^20^. The exclusive abilities of PB to adsorb hydrated Cs^+^ are caused by regular lattice spaces surrounded by cyanide-bridged metals and proton-exchange mechanism acted on the specific Cs^+^ adsorption. The adsorption ability of PB for alkali metal ions increased in the order of Cs^+^ ≫ K^+^ ≥ Na^+^. Moreover, PB has attracted significant attention from both theoretical and applied scientists because of its unique properties and broad range of applications. PB has shown excellent adsorption ability towards Cs and potential for use as an adsorbent. However, PB nanoparticles prepared by precipitation method is usually in the form of very fine powder. Additionally, its long sedimentation time, makes it difficult to separate it from aqueous solution by filtration or centrifugation, necessitating an expensive filtration membrane after Cs adsorption. Nano-sized particles also tend to form agglomerates to minimize their surface energy^21^. In the actual water treatment, adsorbent is required to pack in a column to remove contaminants from water. Powder adsorbents may result in blocking phenomenon, and preparation of bead adsorbents may relieve clogging and post-treatment separation problems. On the other hand, GO has a good supporting material for PB due to the high surface area and surface functional groups. However, GO forms a stable colloidal suspension in water, which makes it difficult to separate from aqueous solution after Cs adsorption^22^. To overcome this issue, an ideal solution is the *in-situ* decoration of PB nanoparticles on a three-dimensional (3D) porous graphene foam, as a support^23^,^24^,^25^,^26^,^27^,^28^,^29^. The 3D graphene foam has a nano-scaled interconnected graphene network with low density, large open pores, and high specific surface area^30^. Considering the excellent properties of PB nanoparticles and graphene foam^31^, a combination might yield enhanced performance.

In this study, PB nanoparticles decorated porous reduced graphene oxide foam (RGOF) composite was prepared *via* a facile hydrothermal method. The formation of the PB/RGOF composite was endorsed by Fourier transform infrared spectroscopy (FT-IR), Raman spectroscopy, and X-ray diffraction (XRD). Transmission electron microscopy (TEM) and scanning electron microscopy (SEM) analyses showed the decoration of cubic PB nanoparticles on the surface of RGOF. The composite was designed to meet the needs of high adsorption and easy separation of radioactive ^137^Cs from contaminated water. The results illustrated that the composite exhibited excellent removal of ^137^Cs from the contaminated water due to the high adsorption capacity of RGOF and good ion-exchange properties of the PB nanoparticles.

## Results

### Mechanism

**Figure 1:**
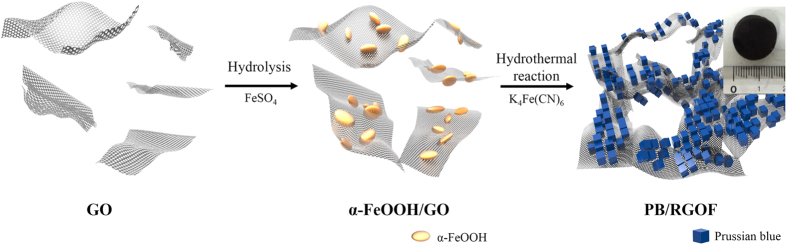
Schematic diagram for the synthesis of PB/RGOF composite.

Figure 1 shows a schematic diagram for the synthesis of the PB/RGOF composite. The mechanism involves three steps. First, GO was dispersed in water because of its strong hydrophilicity and electrostatic repulsion effect^32^. Ferrous sulfate is then added to the GO suspension and stirred vigorously. The ferrous ions tend to diffuse toward the GO sheets *via* electrostatic interactions, which are then oxidized to ferric ions by oxygen containing functional groups present in the GO^33^. The resultant goethite (α-FeOOH, iron oxide/hydroxide) nanoparticles are then deposited on the GO surface *in-situ* by the hydrolysis of ferric ions. Second, potassium ferrocyanide solution is added drop-wise to the above solution. The solution color changes from black to dark blue, indicating the formation of PB nanoparticles. To improve the deposition of PB nanoparticles on the GO surface, the reaction is carried out under acidic condition (pH 3) since Fe^3+^ sites are preferentially created on the GO surface and can then react with the nearby hexacyanoferrate ions. Finally, the solution is sealed in a Teflon-lined autoclave and heated at 180 °C where the GO self-assembles into interconnected networks driven by the combined hydrophobic and π-π stacking interactions, owing to the reduction of GO to RGO^34^,^35^.

### Structural, morphological, surface, and thermal stability studies

**Figure 2:**
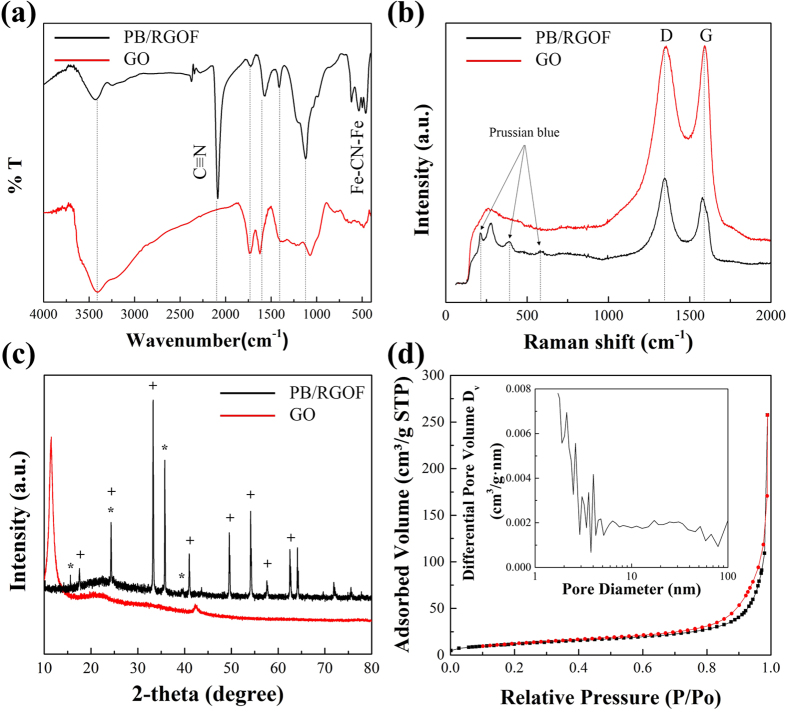
(**a**) FT-IR, (**b**) Raman, (**c**) XRD patterns (peaks corresponding to α-FeOOH (+) and PB nanoparticles (*), respectively) and (**d**) nitrogen adsorption/desorption isotherm of the composite (inset figure is the pore-size distribution).

Figure 2a shows the FTIR spectra of the GO and PB/RGOF composite. The spectrum of GO showed an adsorption band at 1733 cm^−1^, which was assigned to the carboxyl C=O stretching vibration. The band at 3382 cm^−1^ was assigned to the O–H stretching vibration. The adsorption bands at 1615, 1400, and 1052 cm^−1^ were assigned to aromatic C=C, C–OH, and epoxy C–O stretching vibrations, respectively^36^. Compared to GO, the C=O, O–H, and C–O band intensities were reduced dramatically, indicating the successful reduction of GO to RGO. In addition, three vibrational bands located at 2082, 570 and 490 cm^−1^ were ascribed to the stretching vibrations of Fe-CN, Fe-O, and Fe^2+^-CN-Fe^3+^, respectively^37^. The band at 2082 cm^-1^ was due to the presence of a –CN group.

Figure 2b illustrates the Raman spectra of the GO and PB/RGOF composite. For the GO, two prominent bands, D and G were observed at 1310 and 1590 cm^−1^, respectively. The D band represents the disordered carbon arising from structural defects, whereas the G bands corresponds to tangential C–C stretching vibrations^38^. On the other hand, the D and G bands of the composite were shifted slightly. In addition, the characteristic bands observed at 195, 386, and 588 cm^−1^, which were confirmed the existence of PB nanoparticles. The intensity ratio of the D band to G band (*I*_D_/*I*_G_) in the composite (1.079) was higher than that in GO (0.996). These findings demonstrated the formation of a new graphitic domain, i.e., the GO was reduced to RGO after the hydrothermal process.

Figure 2c illustrates the XRD patterns of the GO and PB/RGOF composite. For the GO, a sharp diffraction peak observed at 10.4° was indexed to the (002) plane. In the composite, all the peaks were assigned to the PB nanoparticles and RGO. The diffraction peaks observed at 2θ = 17.4°, 24.8°, 35.3°, and 39.5°, which were indexed to (200), (220), (222), and (400) reflections, respectively, of the face-centered cubic structure of the PB nanoparticles^39^. The (002) plane of graphitic carbon overlapped with the (220) plane of PB nanoparticles. The above results endorsed the formation of PB/RGOF composite.

Figure 2d shows the N_2_ adsorption/desorption isotherm and corresponding BJH pore-size distribution curve of the PB/RGOF composite. Before the measurement, the solid was completely dehydrated by heating at 95 °C overnight under vacuum. This temperature was applied in order to avoid framework collapse. The isotherm revealed that the BET specific surface area of the composite was 43.07 m^2^/g, which was smaller than that of the RGOF (345.24 m^2^/g) (Figure S1). The pore-sizes of the RGOF and PB/RGOF composite were ranged from 2 to 10 nm and 2 to 5 nm, respectively. As shown in Fig. 2d inset, the composite exhibited a pattern closely related to the Type IV adsorption isotherm, according to the IUPAC classification, and showed steep N_2_ gas uptake at realtively low pressure region, indicating the mesoporous nature of the composite.

**Figure 3:**
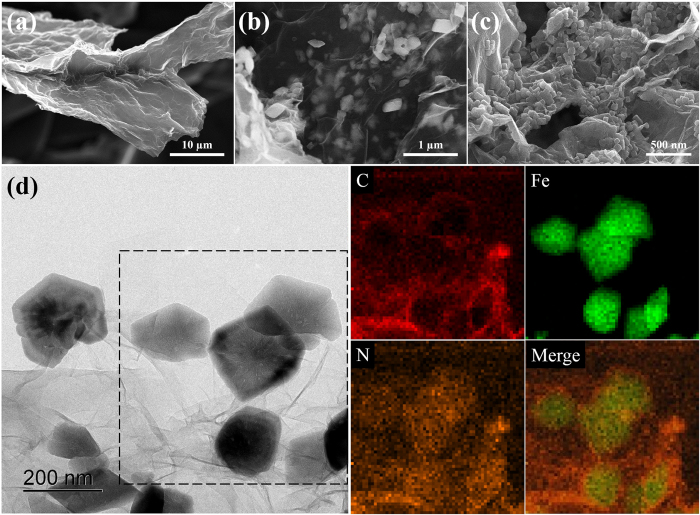
SEM images of the (**a**) GO, (**b**) α-FeOOH/GO, and (**c**) PB/RGOF composite, (**d**) TEM and corresponding EDS mapping of the composite.

Figure 3 presents SEM images of the GO, α-FeOOH/GO, and PB/RGOF composite and TEM image of the composite. The SEM image of GO (Fig. 3a) showed that the nanosheets were not perfectly flat and wrinkled. As shown in Fig. 3b, the GO surface was decorated with ellipsoidal shape α-FeOOH nanoparticles with an average particles size of 300 nm. In the composite (Fig. 3c), a large number of cubic PB nanoparticles of uniform size (50 nm) were decorated on the RGOF surface. As a result of the hydrothermal reaction, the composite exhibited an interconnected 3D network structure with uniformly dispersed pores. As a reference, SEM images of the as-synthesized pure RGOF (without PB nanoparticles) are shown in Figure S2. Figure 3d shows a representative TEM image and corresponding energy dispersive X-ray detector (EDS) mapping of the composite. TEM image clearly showed the decoration of PB particles on the RGOF. EDS mapping analysis indicated the presence of C (from RGO), Fe (from PB), and N (from PB) in the composite.

**Figure 4:**
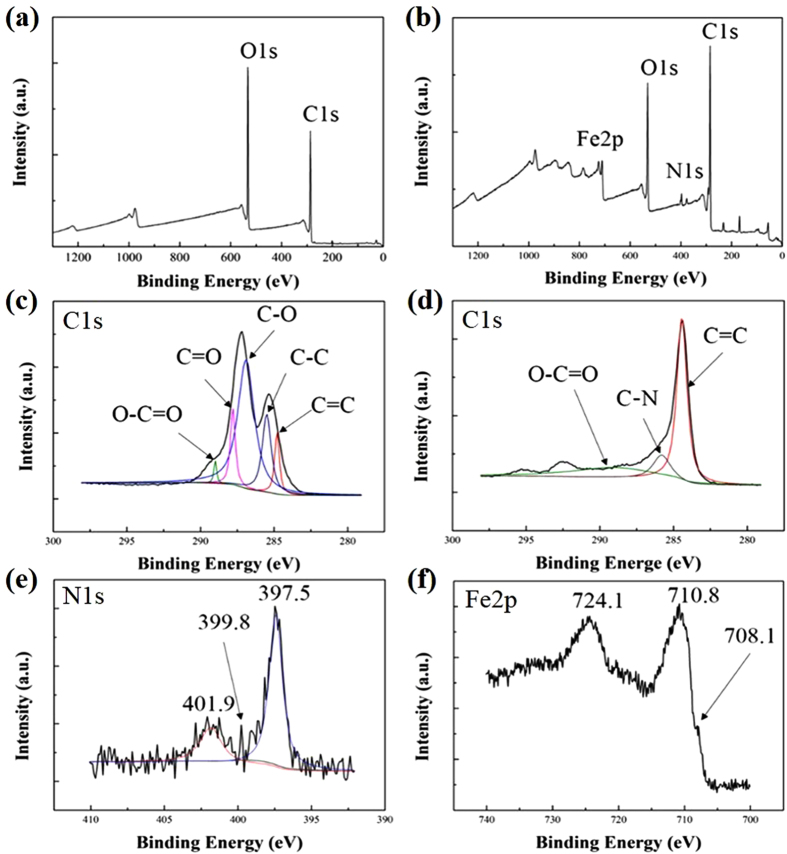
Full scan spectra of the (**a**) GO and (**b**) PB/RGOF composite, (**c**) C1s spectrum of the GO, (**d**) C1s spectrum of the composite, (**e**) N1s spectrum of the composite, and (**f**) Fe2p spectrum of the composite.

Figure 4 demonstrates the survey and core–level spectra of the GO and PB/RGOF composite, respectively. The sharp peaks in the full scan spectra revealed the presence of carbon and oxygen in the GO (Fig. 4a).The survey spectrum of composite (Fig. 4b) exhibited C1s (284.4), O1s (531.4), N1s (397.5), and Fe2p (710.8 eV) peaks, which confirmed the successful decoration of PB nanoparticles on the RGOF surface. The Gaussian fit of C1s core-level spectrum of the GO (Fig. 4c) showed the following five peaks: non-oxygenated C=C (284.8 eV) and C−C (285.5 eV), C–O (286.9 eV), C=O (287.8 eV), and O–C=O (289.0 eV). The C1s spectrum of the composite (Fig. 4d) showed that, compared to GO, the peak intensities of C−O, C=O, and O–C=O were decreased dramatically, indicating the successful reduction of GO to RGO through the hydrothermal process. The core-level spectrum of N1s (Fig. 4e) had three peaks at 401.9, 399.8, and 397.5 eV, signifying the existence of –C≡N in the composite. In the Fe2p spectrum (Fig. 4f), the peaks were observed at 710.8 and 724.1 eV, indicating α-FeOOH, while the peak at 708.1 eV was attributed to [Fe(CN)_6_]^4^,^40^.

**Figure 5:**
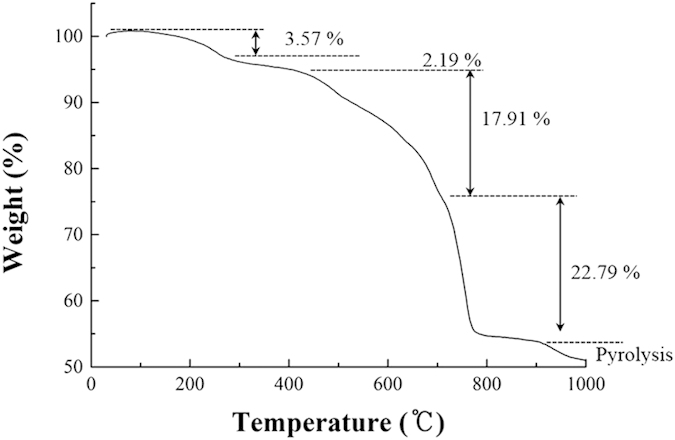
TGA curve of the PB/RGOF composite.

Figure 5 illustrates thermogravimetric analysis (TGA) curve of the PB/RGOF composite at a heating rate of 10 °C/min under nitrogen. The TGA data showed a four-step weight loss. The weight loss (about 3.57%) in the first step below 200 °C is attributed to the loss of residual moisture. The second minor weight loss step (about 2.19%) at above 300 °C corresponds to the loss of oxygen functional groups present in the RGO. The third step between 400–750 °C was attributed to the decomposition of the cyano groups^41^,^42^. The fourth major weight loss at above 700 °C was ascribed to the combustion of RGO. Pyrolysis of the carbon skeleton was observed at around 900 °C. Based on the TGA data, the amount of PB nanoparticles present in the composite was found to be 33.17%, which was calculated from the decomposition percentage of cyano groups (17.91%).

### Removal efficiency of the adsorbent

**Figure 6:**
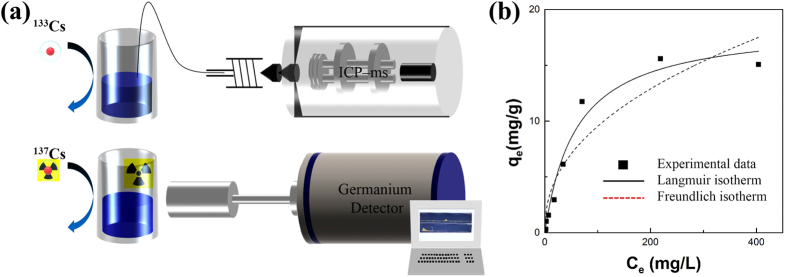
(**a**) Schematic diagram for the measurement of Cs concentration (inactive ^133^Cs and radioactive ^137^Cs) using an ICP-MS and germanium detector and (**b**) experimental data fitted with Langmuir and Freundlich adsorption isotherm models.

The adsorption capacity of the RGOF and PB/RGOF composite as a function of inactive ^133^Cs concentration was investigated by varying the initial concentration from 1 to 500 ppm. Batch experiments were carried out with the Cs concentration of 200.69 ppb containing 10 mg of the adsorbent. After shaking the vial for 12 h, the aqueous solution was removed by filtration. The results (Table 1) showed that the Cs removal efficiency of RGOF and composite was found to be 50.72 and 94.17%, respectively. Even though the surface area of the composite was much smaller than that of RGOF, the distribution coefficient (*K*_*d*_) value of the composite (6455.34 mL/g) was 15 times higher than that of RGOF (411.77 mL/g). This may be attributed to the high affinity PB nanoparticles towards Cs. The coefficient *K*_*d*_ expresses the chemical binding affinity of the target metal-ion to an adsorbent, and is most meaningful at dilute concentrations, and the higher *K*_*d*_ value indicating stronger binding affinity. Generally, a *K*_*d*_ value of 5000 is considered good^43^. The schematic diagram for the measurement of Cs (inactive ^133^Cs and radioactive ^137^Cs) using the ICP-MS and germanium detector is shown in Fig. 6a.

Although, few reports have been published concerning the Cs^+^ adsorption using the PB particles^41^,^44^, the intrinsic Cs^+^ adsorption mechanism of the PB nanoparticles is still unclear. The chemical/physical interaction has been commonly accepted that the exclusive ability of PB to adsorb hydrated Cs^+^ are caused by the regular lattice spaces surrounded by cyanide-bridged metals (physical adsorption) and proton-exchange mechanism acted on the specific Cs^+^ adsorption (chemical adsorption). The adsorption ability of the PB for alkali metal ions is influenced by their radii as hydrated ions. The radii of the alkali metal ions are in the order of Cs^+^ (1.19) < K^+^ (1.25) < Na^+^ (1.84 A^°^) and the smaller radius of Cs^+^ was probably fitted in the PB lattice spaces^44^. It is well known that, due to a very similar hydration radius, K^+^ can compete very well with Cs^+^ in binding to PB. In the proton-exchange mechanism, Cs^+^ ions would be efficiently adsorbed throughout the crystal lattice spaces apart from strong electrostatic attraction^41^.

### Adsorption isotherm

Adsorption isotherm models are widely used to describe the adsorption progress and investigate the mechanism of adsorption. Adsorption isotherms are basic requirements for the design of adsorption system. Adsorption equilibrium data provide information on the capacity of adsorbent on the amount required to remove a unit mass of pollutant under the system conditions. Figure 6b shows that the adsorption capacity increased rapidly with increasing the inactive ^133^Cs concentration up to 200 ppm, which may be attributed to the fact that sufficient active sites were available. At higher Cs concentrations, competition for available adsorption sites were decreased, resulting in a decrease in the adsorption capacity. The Langmuir and Freundlich adsorption isotherm models were applied to fit the adsorption data at equilibrium^45^,^46^. The Langmuir model is valid for monolayer adsorption under the assumption that all binding sites are free. The nonlinear form of the equation is written as:


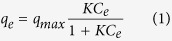


where *q*_*e*_ and *q*_*max*_ are the equilibrium adsorption capacity and monolayer maximum adsorption capacity (mg/g), respectively. *K* is a constant related to the affinity between the adsorbent and adsorbate. The Freundlich adsorption isotherm model is considered to be an empirical equation that describes multi-layer adsorption with several types of adsorption sites on the surface of the adsorbent. The equation is in the following form:


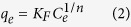


where *K*_*F*_ and *n* are the Freundlich constants relative to the multilayer adsorption capacity. Figure 6b shows the experimental data fitted with the Langmuir and Freundlich models. The isotherm was well-fitted by the Langmuir model with an R^2^ value of 0.97. The fitted parameters and correlation coefficient for both models are listed in Table 2. The maximum adsorption capacity of the PB/RGOF composite was determined to be 18.67 mg/g.

### Radioactive ^137^Cs decontamination

Various amounts of the PB/RGOF composite (0.1–1.0 mg/mL) were added to the radioactive ^137^Cs solution and shaken for 12 h. The composite was then separated from the solution by filtration, after which the solution was analyzed to determine the ^137^Cs concentration. Performance parameters including the removal efficiency of ^137^Cs and decontamination factor (DF) were obtained and summarized in Table 3. The ^137^Cs removal reached 95.5% using 0.5 mg/mL of the composite, which was attributed to the large number of adsorption sites. Further increases in the adsorbent dosage to 1 mg/mL, the removal efficiency was increased to 99.5%. Therefore, the optimal dosage for ^137^Cs removal was 0.5 mg/mL of the composite. In addition, the high DF (>20) further confirmed the potential application of the PB/RGOF composite for the decontamination of ^137^Cs containing radioactive water.

## Discussion

In summary, we have successfully fabricated the PB nanoparticles decorated RGOF composite *via* a facile one-step hydrothermal method for efficient removal of radioactive ^137^Cs. The SEM and TEM analyses showed that the cubic PB nanoparticles of uniform sizes were decorated on the porous RGOF surface. The excellent adsorption performance towards Cs may be attributed to the large surface area of the 3D porous foam and good ion-exchange properties of the PB nanoparticles, which can replace Cs ions by using potassium ions. The adsorption results revealed that the removal efficiency of ^137^Cs from the radioactive water was 99.5%. The adsorption isotherm was fitted-well by the Langmuir model with a maximum adsorption capacity of 18.67 mg/g. The high DF factor (213.39) confirmed the successful removal of ^137^Cs from the radioactive water. This synthetic procedure could be extended to the preparation of a wide range of 3D hierarchical graphene framework/inorganic material composites for potential applications in various fields, particularly in environmental applications.

## Methods

### Chemicals

Graphite powder, potassium persulfate, phosphorous pentoxide, potassium permanganate, ferrous sulfate, potassium ferrocyanide, H_2_SO_4_, and HCl were obtained from Sigma-Aldrich. Inactive Cs solution (KANTO Chemical Co. Inc.) and radioactive cesium (^137^Cs) were obtained from the Korea Atomic Energy Research Institute (KAERI). All other chemicals were of analytical grade and used without further purification.

### Caution

Radioactive cesium is an extremely hazardous chemical; it should be prepared and handled by trained professionals in a designated space. Individuals handling this material should wear a facemask, gloves, and a protective suit.

### Synthesis of Prussian blue/reduced graphene oxide foam composite

GO was prepared by chemical exfoliation of graphite powder based on a modified version of Hummer’s method^47^. In a typical experiment, the GO suspension was prepared by dispersing 70 mg of GO in 20 mL water *via* ultrasonication. The pH of the suspension was adjusted to 3. Ferrous sulfate (1 mmol) was then added and stirred. Ferrous ions were oxidized to ferric ions followed by hydrolysis to form α-FeOOH nanoparticles. Finally, 10 mM potassium ferrocyanide solution was added and stirred continuously, during which time the color of the mixture gradually changed from brown to dark blue, suggesting the formation of PB nanoparticles. The resultant mixture was subsequently transferred to a Teflon-lined reactor, and the reaction was conducted at 180 °C for 12 h. The obtained composite foam was separated and washed with distilled water several times by centrifugation. Finally, it was freeze-dried at −80 °C for 12 h.

### Characterization

SEM images were taken using an S-4800SE microscope at an acceleration voltage of 15 kV. FT-IR spectra were recorded using a Jasco FT/IR-6600. X-ray diffraction (XRD) patterns were collected using a Bruker D2 phaser (Germany) diffractometer with Cu Kα radiation. Nitrogen adsorption/desorption isotherms were obtained at 77 K on an ASAP 2010 apparatus. TEM analysis was performed using a Tecnai G2, (FEI, Netherland) microscope at an accelerating voltage of 200 kV, equipped with an EDS. X-ray photoelectron spectroscopy measurements were obtained using a Thermo Scientific, K–Alpha electron spectrometer with an Al X–ray source. Raman spectra were determined using a 532 nm laser Raman microscope (UniRAM, UniNanoTech., Korea). TGA was performed using a Tarsus^®^ TG 209 F3 from room temperature to 1000 °C at a heating rate of 10 °C/min under nitrogen atmosphere.

### Adsorption experiment

Batch experiments were carried out in a 10 mL vial with 5 mL of the inactive Cs solution (200.69 ppb) containing 10 mg of the PB/RGOF composite. After shaking the vial for 12 h, the aqueous solution was removed and passed through a syringe type filter. The initial and residual Cs concentrations were analyzed using an inductively coupled plasma mass spectrometer (ICP-MS, PerkinElmer ELAN 6100). The distribution coefficient (*K*_*d*_) was defined to evaluated the ability of the adsorbent to be removed from the contaminated water:


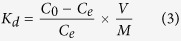


where *C*_*0*_ and *C*_*e*_ represent the initial and equilibrium concentration of Cs, respectively. *V* denotes the volume of Cs solution and *M* is the mass of the adsorbent. The experiment was performed in triplicate and the mean value was reported.

### Adsorption isotherm

The adsorption isotherm was investigated based on batch experiments. Inactive Cs (^133^Cs) was used to study the adsorption behavior. The initial Cs concentration was varied from 1–500 ppm. The required amount of adsorbent (10 mg) was added to the Cs solution (4 mL). The vial was then shaken at 60 rpm on a rotary shaker for 12 h. After equilibrium, the adsorbent was separated by filtration and the residual Cs concentration was analyzed using the ICP-MS.

### Decontamination of ^137^Cs solution

The solutions containing radioactive ^137^Cs was prepared by diluting a stock solution to approximately 100 Bq/g. The required amount of adsorbent (1, 5, and 10 mg) was dispersed in a 10 mL ^137^Cs solution, after which the vial was shaken for 12 h. The aqueous solution was then filtered through a syringe type filter and the adsorption capacity was measured using an HPGe detector (Canberra, USA). The removal efficiency (%) and decontamination factor (DF) values, which were defined by the following equation to assess the adsorption capacity of the PB/RGOF composite towards ^137^Cs:


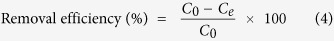



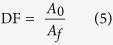


where *C*_*0*_ and *C*_*e*_ represent the initial and equilibrium concentrations of the Cs, respectively, and *A*_*0*_ and *A*_*f*_ are the cesium radioactivity in the initial and final solutions after treatment, respectively.

## Additional Information

**How to cite this article**: Jang, S.-C. *et al.* Porous three-dimensional graphene foam/Prussian blue composite for efficient removal of radioactive ^137^Cs. *Sci. Rep.* **5**, 17510; doi: 10.1038/srep17510 (2015).

## Supplementary Material

Supplementary Information

## Figures and Tables

**Table 1:** Removal efficiency of ^133^Cs (%) and *K*_*d*_ value.

Material	*C*_*0*_(ppb)	*C*_*e*_(ppb)	Removalefficiency (%)	*K*_*d*_(mL/g)
RGOF	200.69	98.89	50.72	411.77
PB/RGOF	200.69	11.71	94.17	6455.34

**Table 2:** Isotherm parameters for the adsorption of ^133^Cs using PB/RGOF composite.

Langmuir model			Freundlich model		
*K*_*L*_(L/mg)	*Q*_*m*_(mg/g)	R^2^	*K*_*F*_(L/mg)	*n*	R^2^
0.017	18.67	0.97	1.29	2.3	0.89

**Table 3:** Adsorption parameters of radioactive ^137^Cs using PB/RGOF composite.

Concentration of PB/RGOFcomposite (mg/ml)	Activity beforetreatment (Bq/g)	Activity aftertreatment (Bq/g)	Removalefficiency (%)	DF
0.1	88.70	10.91	87.70	8.13
0.5	90.14	4.10	95.45	21.98
1.0	87.49	0.41	99.53	213.39
