# Supplementary material for: Porous three-dimensional graphene foam/Prussian blue composite for efficient removal of radioactive 137Cs
Source: Sci Rep. 2015 Dec 16;5:17510. doi: 10.1038/srep17510 (PMC4680859; doi:10.1038/srep17510)
Supplement: Supplementary Information [file srep17510-s1.pdf]

Supporting Information

Porous three-dimensional graphene foam/Prussian blue composite for efficient removal of radioactive ^{137}Cs

Sung-Chan Jang, Yuvaraj Haldorai, Go-Woon Lee, Seung-Kyu Hwang, Young-Kyu Han, Changhyun Roh*, and Yun Suk Huh*

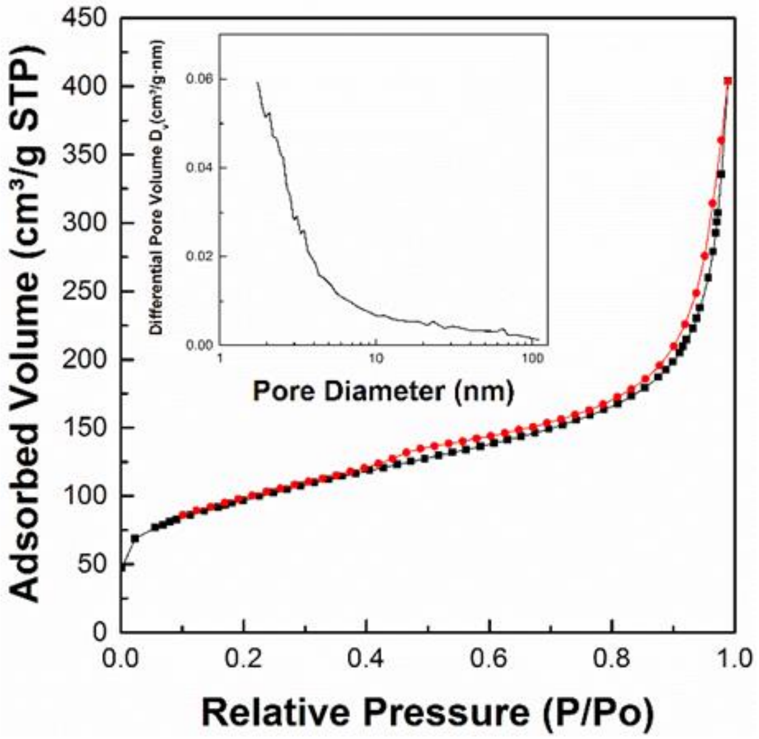

Figure S1. Nitrogen adsorption/desorption isotherm of RGOF.

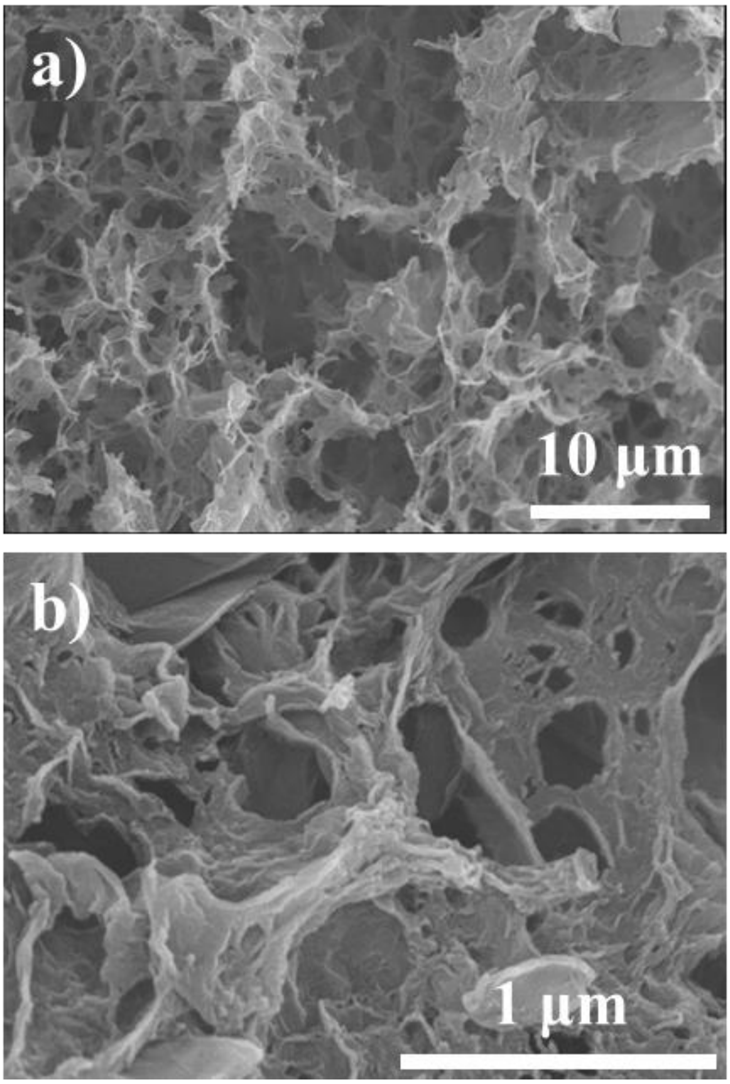

Figure S2. SEM images of the RGOF (different magnifications).
